# Active forgetting and neuropsychiatric diseases

**DOI:** 10.1038/s41380-024-02521-9

**Published:** 2024-03-26

**Authors:** Jacob A. Berry, Dana C. Guhle, Ronald L. Davis

**Affiliations:** 1 Department of Biological Sciences, University of Alberta, Edmonton, AL T6G 2E9 Canada; 2 Department of Neuroscience, UF Scripps Institute for Biomedical Innovation & Technology, 130 Scripps Way, Jupiter, FL 33458 USA

**Keywords:** Neuroscience, Psychiatric disorders

## Abstract

Recent and pioneering animal research has revealed the brain utilizes a variety of molecular, cellular, and network-level mechanisms used to forget memories in a process referred to as “active forgetting”. Active forgetting increases behavioral flexibility and removes irrelevant information. Individuals with impaired active forgetting mechanisms can experience intrusive memories, distressing thoughts, and unwanted impulses that occur in neuropsychiatric diseases. The current evidence indicates that active forgetting mechanisms degrade, or mask, molecular and cellular memory traces created in synaptic connections of “engram cells” that are specific for a given memory. Combined molecular genetic/behavioral studies using *Drosophila* have uncovered a complex system of cellular active-forgetting pathways within engram cells that is regulated by dopamine neurons and involves dopamine-nitric oxide co-transmission and reception, endoplasmic reticulum Ca^2+^ signaling, and cytoskeletal remodeling machinery regulated by small GTPases. Some of these molecular cellular mechanisms have already been found to be conserved in mammals. Interestingly, some pathways independently regulate forgetting of distinct memory types and temporal phases, suggesting a multi-layering organization of forgetting systems. In mammals, active forgetting also involves modulation of memory trace synaptic strength by altering AMPA receptor trafficking. Furthermore, active-forgetting employs network level mechanisms wherein non-engram neurons, newly born-engram neurons, and glial cells regulate engram synapses in a state and experience dependent manner. Remarkably, there is evidence for potential coordination between the network and cellular level forgetting mechanisms. Finally, subjects with several neuropsychiatric diseases have been tested and shown to be impaired in active forgetting. Insights obtained from research on active forgetting in animal models will continue to enrich our understanding of the brain dysfunctions that occur in neuropsychiatric diseases.

## Introduction

**Fig. 1:**
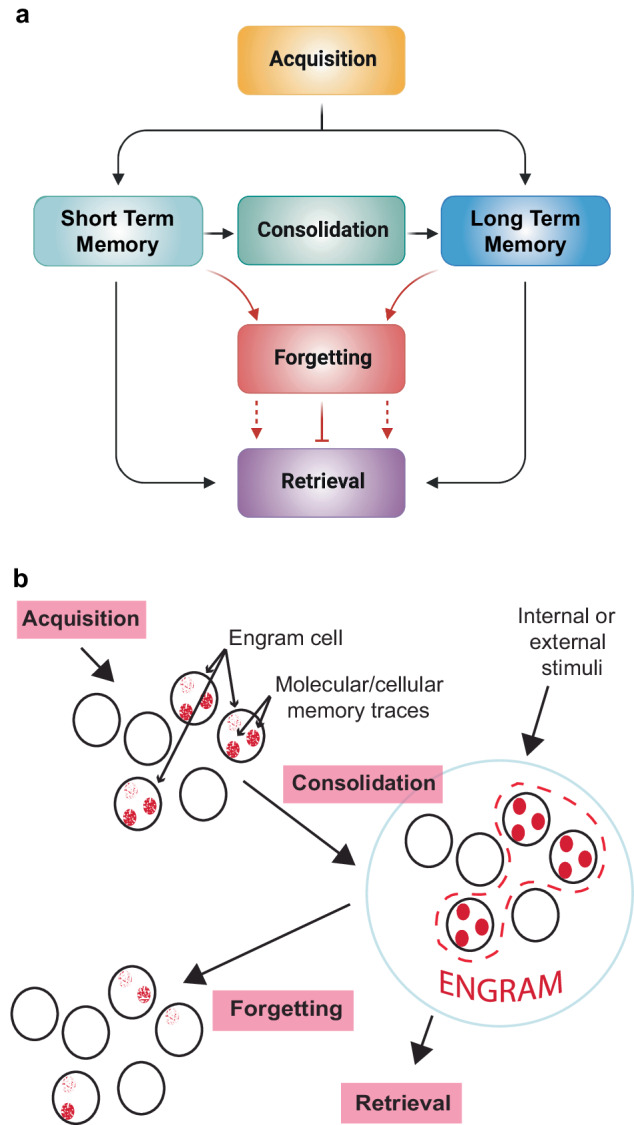
Principal memory operations and cellular events. **a** The nervous system uses four operations for short- and long-term memory formation: Acquisition, Consolidation, Forgetting, and Retrieval. Acquisition is synonymous with “learning,” and represents the initial encoding of information. Consolidation refers to the processes involved in stabilizing memory over time. Forgetting involves mechanisms whereby memories can be erased or hidden from retrieval. Retrieval is simply the recollection, or recall, of existing memories. **b** Cartoon illustrating the broad cellular and network events of memory formation. During acquisition, selected cells in the nervous system undergo molecular or biochemical changes that alter their physiological state. These selected cells are known as engram cells, and the molecular or biochemical changes within the engram cells are termed molecular or cellular memory traces. Consolidation mechanisms stabilize the cellular memory traces and the selected engram cells. The engram cells and their corresponding memory traces, together, represent the overall “engram” for a given memory. The activity of forgetting cells can erode the memory traces and cause memory failure.

A normally functioning human brain has the extraordinary ability to acquire and store a countless number of memories that form each day. This is accomplished by the network of ~86 billion neurons and at least as many nonneuronal cells that make up the human brain [1]. The broad process of memory formation, storage, and use occurs through at least four operations: acquisition (or learning), consolidation, retrieval, and forgetting (Fig. 1a). Memory acquisition alters the physiological state of selected and sparse neurons in ways that generate a neural code for each memory (Fig. 1a,b). The alterations in physiological state, which are broadly termed as molecular and cellular memory traces, can include any change in the cellular activity of the cell induced by learning that becomes part of its neural code. Such memory traces include altered expression or function of ion channels that change the excitable state of neurons so that electrical signaling is increased or decreased. They include altered intracellular signaling pathways that influence the neuron’s overall ability to integrate inputs from different types of cues, and synaptic changes that influence the neuron’s ability to stimulate synaptic partners. They include neuronal growth processes that establish new connections, or neurite retraction to remove existing connections. Some of the molecular and cellular memory traces support only short-term memory (STM), and others are further processed by protein-synthesis dependent consolidation mechanisms leading to persistent and resilient long-term memory (LTM). Collectively, the molecular and cellular memory traces induced by learning across all neurons engaged by the learning event together comprise the memory engram (Fig. 1b) that can guide behavior upon subsequent retrieval [2–5].

The fate of the engram depends on the fate of these molecular and cellular memory traces formed at acquisition. One fate leads to consolidation, the processes that stabilize a memory so that it is long-lived and resistant to insults like electroconvulsive shock and inhibition of protein synthesis (Fig. 1a, b). Past neuroscience research has focused nearly entirely on this operation and on acquisition [2, 6–15]. Indeed, one might point to the discovery of long-term potentiation by Bliss and Lomo in 1973 [16] as the start of research on acquisition, and the discovery in the 1960s by Flexner and Flexner, Agranoff, and others, that inhibitors of protein synthesis block memory consolidation as the start of research on consolidation [17]. Although much has been learned about acquisition and consolidation across the last 5-6 decades, the neuroscience understanding of retrieval remains largely theoretical [18], but with some mechanistic studies suggesting that retrieval occurs from internal or external stimuli that activate the same engram cells that are selected during acquisition (Fig. 1). Mechanistic studies of forgetting began only about one decade ago, so there remains much more to be discovered. Nevertheless, the mechanisms underlying forgetting are integral to many different brain disorders, including those in neuropsychiatry.

## Forgetting and neuropsychiatric diseases

Broad memory dysfunction occurs in many different psychiatric diseases [19, 20]. This indicates that the neurobiological mechanisms serving one or more of the four operations underlying memory formation (Fig. 1a) are disrupted at the cellular and/or network level, perhaps in different ways and degrees in the different diseases. And it may be that operations underlying forgetting are particularly vulnerable in some psychiatric diseases and not others. But disentangling an impairment in forgetting from the other operations underlying memory (Fig. 1a) is not a trivial task. For instance, the impairment in the ability to hold information “on-line” across a short period of time to accomplish a working memory task in individuals with schizophrenia [21] could be due to ineffective encoding of the information or overactive forgetting. An air-tight assignment to processes underlying forgetting in this case would require knowing that all cellular memory traces in all engram neurons are formed to the same strength and that retrieval mechanisms are identical between diseased and control individuals. This, of course, is currently impossible. Nevertheless, experimental psychologists have devised memory tasks that provide relatively pure measurements for the operation of forgetting [22]. In addition, the nature of memory impairments observed in some neuropsychiatric diseases fits rather precisely with problems in the operation of forgetting, rather than acquisition, consolidation, or retrieval.

Intrusive memories, distressing thoughts, and unwanted impulses are common in many neuropsychiatric disorders. These may occur from the involuntary recall of unwanted thoughts and memories, due to the failure to diminish or erase the memories through forgetting processes [23]. For instance, post-traumatic stress disorder (PTSD) occurs from traumatic and often life-threatening events, with the persistent and reoccurring memory of these events disrupting the lives of individuals with the disorder [24]. This can be explained as due to impaired forgetting. Such enhanced memory is also a feature of addiction, where addiction-associated cues take on enhanced salience compared to more pedestrian cues [25]. Furthermore, it may be that impaired forgetting plays a part in other psychiatric disturbances, including the unwanted maintenance of a misperception that could lead to a distorted interpretation of reality, that is, the generation of a delusion [26].

Tests of individuals with such disorders have revealed impairments in two classes of experimentally measurable forgetting, intentional and incidental forgetting (Table 1). Intentional forgetting occurs through effortful suppression of an unwanted memory at the time of retrieval [27]. One method for measuring intentional forgetting involves the Think/No-Think (TNT) paradigm [22]. Subjects are first presented with cue-target pairs, such as word pairs, until they can recall the targets upon presentation of the cue. In a second phase, the subjects are repeatedly presented with the cues and specific instructions to think about the target for some of the cues, or to repress thoughts about the target for other cues. The final test then measures forgetting for all cue-target pairs, with normal subjects recalling No-Think targets less frequently than Think targets. Incidental forgetting occurs when the selective retrieval of some memory items reduces the recall of unretrieved competing items [22]. This can be measured by having subjects learn multiple items within a category, such as Sports/Basketball, Sports/Soccer, Sports/Tennis, Sports/Swimming, etc., followed by practice in retrieving a subset of the items within the category. The retrieval of practiced items promotes the unintentional forgetting of the non-practiced items. Thus, a reduced potency of intentional or incidental forgetting mechanisms, or other mechanisms for forgetting, can account for the unwanted and intrusive thoughts and memories in the psychiatric diseases listed in Table 1.

**Table 1:** Deficits in two broad classes of active forgetting measured in subjects with neuropsychiatric disorders.

Disorder	Impairment	References
Addiction	Incidental forgetting	[148]
Anxiety	Intentional and incidental forgetting	[28, 148–150]
Attention-Deficit/Hyperactivity Disorder (ADHD)	Incidental forgetting	[151, 152]
Depression	Intentional and incidental forgetting	[28, 148, 153]
Obsessive-Compulsive Disorder (OCD)	Intentional forgetting	[28]
Post-Traumatic Stress Disorder (PTSD)	Intentional and incidental forgetting	[28]
Schizophrenia	Intentional and incidental forgetting	[28, 154–156]

Accumulating evidence indicates that intentional and incidental forgetting occurs partly at the network level, with selective memory retrieval or retrieval suppression activating the lateral prefrontal cortex which promotes inhibition in other brain regions that contain the engram cells of memories to be diminished [22, 28]. However, model organism research into forgetting processes shows that forgetting also involves cellular and molecular mechanisms. These mechanisms are the focus of the remainder of this review.

## Neurobiology of active forgetting

To accelerate and deepen our understanding of forgetting in humans, researchers are using genetically tractable model organisms to understand the underlying genes, circuits, and cellular processes. While model research into forgetting remains in early stages, in little over a decade, researchers have already utilized a variety of model organisms from mammals to invertebrates, including *Drosophila melanogaster* (fruit fly) and *Caenorhabditis elegans* (worms), to ask fundamental questions about forgetting. Currently, *Drosophila* has been exceptionally successful, relying on its superb balance of a simple nervous system, complex behaviors, and genetic expediency.

Rapid progress in understanding the genetic and cellular mechanisms of forgetting in *Drosophila* can, in part, be attributed to large scale memory suppressor screens. These screens identify genes that, when disrupted, improve memory performance, and thus normally “suppress” memory. Recent screens involving thousands of genes have identified many memory suppressor genes, some of which specifically disrupt forgetting but leave learning intact [29–31]. These studies have utilized the most well-studied type of *Drosophila* memory, wherein the flies learn to associate a specific odor with either rewarding or punishing stimuli. This type of associative learning relies on responses of the olfactory system to the odor (the conditioned stimuli, CS) integrated with signaling from dopaminergic neurons (DAn) responding to the reinforcing stimuli (the unconditioned stimulus, US). Importantly, findings using this associative memory paradigm have uncovered many similarities to mammalian memory systems including conserved roles for cAMP, CREB and dopamine [32].

Forgetting research in model systems is expanding quickly and many significant findings have already been made that give insight into how our brains forget, discussed below. In this section, we will first discuss the cellular mechanisms that underlie how engrams are altered by forgetting, including dopamine neuron regulation of engram cell synapses, actin cytoskeletal remodeling, and glutamatergic receptor endocytosis. We will subsequently discuss how forgetting is regulated at the network level by neural and glial cells, and how this allows external and internal factors to tune forgetting.

### DA neuron modulation of engram synapses

To understand forgetting, it is helpful to know how memories are formed in the first place. Memories are acquired in the brain as changes in the synaptic input or output of memory specific “engram cells” [5]. In *Drosophila*, memory engrams are thought to be primarily encoded within the mushroom body (MB) brain structure as it is central to acquiring, storing, and retrieving visual and olfactory memories [32, 33]. The foundation of the MB is a network of ~2000 MB intrinsic neurons per hemisphere (or MBn) wherein sparse and specific sets of these neurons respond to and encode information about different sensory stimuli including olfactory, visual, and gustatory [34, 35]. MBn axons synapse heavily onto an array of output neurons (MBOn) and these synaptic connections have been shown to be critical sites for memory storage [36, 37]. Thus, the MBn and MBOn function as “engram cells” as their shared synaptic connections are modified to encode memories as a “synaptic engram”.

What modifies MBn:MBOn synapses to encode memories? A variety of neurons whose axons project and connect heavily with MBn:MBOn synapses [38] express Tyrosine Hydroxylase, the rate-limiting enzyme in dopamine (DA) production and thus they are referred to as dopamine neurons (DAn). Blocking broad DAn synaptic output or DA receptor expression in the MBn completely blocks learning [39, 40], whereas artificial stimulation of DAn in the presence of odor is sufficient to form olfactory memory [41, 42]. Therefore, the DAn transmit the US information during learning. In fact, distinct DAn are activated by specific stimuli that have value to the animal, including sugar rewards or punishments like electric shock and noxious temperature [38, 43]. During learning, US driven DAn release converges with the activation of a sparse set of MBn:MBOn synapses specific to the odor. This drives specific synaptic changes that alter the connectivity between odor specific MBn and distinct MBOn and allow altered behavioral responses to the odor after learning [33, 36, 37, 44–46]. These DAn mediated memory traces can take the form of synaptic depression [36, 37] or potentiation [46, 47] of odor specific MBn:MBOn synapses, resembling long-term depression (LTD) and potentiation (LTP) in mammals, and can last from hours [36, 37] or days [46, 47]. Therefore, in *Drosophila*, DA neurons are critical for encoding memory engrams by altering synaptic strength of engram cells.

**Fig. 2:**
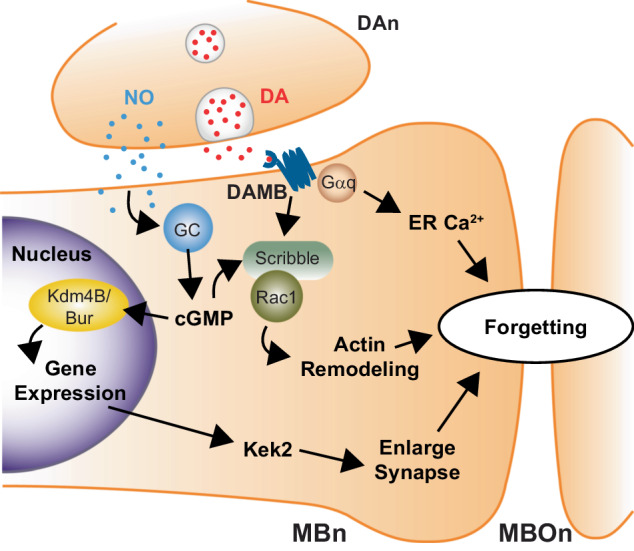
Dopamine neuron mediated active forgetting pathways in *Drosophila*. Dopamine neurons (DAn) modulate forgetting by driving independent active forgetting pathways within engram synapses (MBn:MBOn) using co-transmitters NO and DA. DA signaling through the MBn expressed DAMB receptor drives forgetting through two fundamental pathways: 1) coupling to Gαq to drive ER Ca2+ release in MBn synapses, or 2) signaling through Scribble/Rac1 complex to regulate actin remodeling. In parallel, DAn terminals synthesize NO gas that diffuses into MBn and binds the Guanylyl Cyclase (GC, or GycB100) and generates cGMP. NO mediated effects require Scribble. DAn->NO->cGMP signaling also drives gene-expression based forgetting through co-localization of Kdm4B/Bur to genomic sites. Kdm4B/Bur activity drives expression of many genes and enlargement of MBn synapses, possibly through Kek2 expression.

As it turns out, the same DAn that encode memories are also active after learning and are central regulators of forgetting (Fig. 2) [48–50]. Reactivation of DAn after the flies are trained is sufficient to cause forgetting, whereas blocking synaptic output prolongs memory retention [48, 49, 51]. During learning DA release drives acquisition through one DA receptor (dDA1), but after learning DA signals through another receptor DAMB for forgetting [40, 48]. For example, flies mutant for DAMB have relatively normal learning but memory decay is significantly reduced, whereas overexpression of DAMB in the MBn accelerates forgetting [48, 52]. At a physiological level, dDA1 receptor signaling during learning drives synaptic depression between odor specific MBn:MBOn synapses, whereas DAMB signaling is required for potentiating these connections [53]. Therefore, these results indicate that DA based forgetting occurs by re-potentiating previously depressed engram synapses caused by learning [37]. However, it remains to be seen if the re-potentiation occurs through reversing the specific mechanism of depression or if the depression is masked by independent potentiation mechanisms in the same synapses.

Several different cellular signaling mechanisms have been identified that transmit DA mediated forgetting signals downstream in MBn to affect synaptic engrams (Fig. 2). Interestingly, unlike the learning DA receptor dDA1, the DAMB receptor couples strongly and uniquely to Gαq, causing IP3-dependent calcium efflux from the endoplasmic reticulum (ER) [53, 54]. This DAMB→Gαq coupling is likely an important driver of forgetting as reduction of Gαq in MBn reduces forgetting of STM [54]. Furthermore, physiological studies indicate that DAMB signaling through Gαq robustly drives ER Ca^2+^ release in MBn resulting in MBn:MBOn synaptic potentiation required for memory flexibility [53]. Thus, current data suggest that DAMB receptor signaling utilizes Ca^2+^ signaling from the ER to modulate engram synapse strength and cause forgetting. It remains unclear what connects Ca^2+^ release from the ER to synaptic potentiation in the MBn. However, it is notable that the ER in mammals stores Ca^2+^ in recently activated neurons and regulates synaptic Ca^2+^ and neural transmission [55]. For example, ER Ca^2+^ release at the presynaptic terminal is a major driver of evoked neurotransmission and regulator of synaptic plasticity in hippocampal synapses [56, 57]. Therefore, one interesting possibility is that learning leads to Ca^2+^ storage within the ER of odor activated MBn synapses and DA→DAMB→Gαq signaling then leads to release of this Ca^2+^ to potentiate synaptic release and cause forgetting.

Another important component of DA→DAMB mediated active forgetting involves the scaffolding protein Scribble, which was discovered in a large memory suppressor screen [30, 52]. Scribble is expressed in MBn and while dispensable for learning, is critical for both memory decay and retroactive interference-based forgetting [52]. Scaffolding proteins, like Scribble, function to assemble different components in a signaling cascade. Interestingly, Scribble was shown to function downstream of DA→DAMB signaling and to interact with the small GTPase Rac1 to mediate cytoskeleton remodeling-based forgetting, which we will discuss in more detail in the next section. At the physiological level, Rac1 activity is required for the decay of MBn:MBOn synaptic depression underlying STM [58]. Therefore, Scribble connects DA→DAMB mediated signaling at the plasma membrane with downstream cytoskeleton remodeling to drive forgetting through re-potentiation of depressed engram synapses (Fig. 2). The mechanism by which Scribble facilitates this intracellular communication and whether it also scaffolds Gαq and DAMB remains unknown, but it clearly functions as an important signaling nexus for forgetting. Future studies to uncover other signaling components it scaffolds will be revealing.

While DA is central to modulating forgetting of MBn:MBOn engrams, it was recently discovered that DAn also release nitric oxide (NO) gas as a co-transmitter that strongly modulates forgetting [50]. Remarkably, this study demonstrated that during learning the DA neurons release two signals: 1) DA that acts through dDA1 to immediately depress MBn:MBOn engram synapses and encode memory, and 2) NO which binds to the NO receptor Guanylyl Cyclase GycB100 (GC) expressed in the MBn to stimulate cGMP production. A role of GycB100 in memory enhancement, reported by an earlier memory suppressor screen, supports this model [30]. Interestingly, learning induced NO signaling does not affect the acquisition of STM but instead leads to a delayed process that accelerates forgetting over time. This forgetting allows better memory flexibility so that flies can adapt to new associations. Mechanistically, the data suggest NO signaling promotes forgetting by re-potentiating MBn:MBOn engram synapses and interfering with DA mediated synaptic depression, similar to DA→DAMB mediated forgetting. Additionally, NO mediated effects require Scribble in the MBn. Future experiments are needed to confirm the physiological effects of NO on synaptic plasticity and investigate the relationship between NO and DAMB and actin remodeling-based forgetting. Regardless, a single learning event can drive opposing cellular traces in the same synapses, one that supports the memory and one that suppresses it later and allows future memory flexibility.

**Fig. 3:**
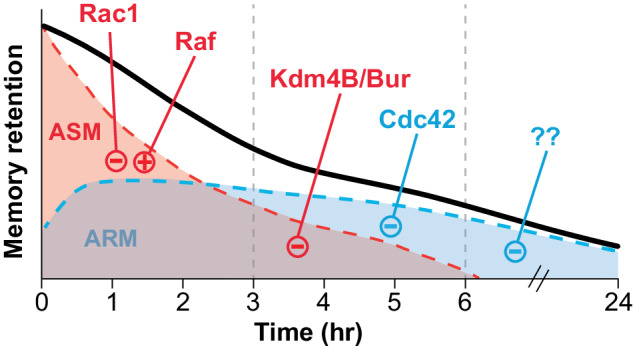
Distinct signaling regulates forgetting of different memory phases and types in *Drosophila*. A single trial of aversive olfactory conditioning yields a short-lived memory that is labile and anesthesia-sensitive (ASM) and a consolidated form of memory that is longer-lasting and anesthesia-resistant (ARM). Retention of STM (black line) and its ASM (red) and ARM (blue) components over time require distinct and sequentially engaged cellular pathways. Upon learning, labile ASM is formed and within 1 hour a consolidated ARM component forms. The forgetting of ASM proceeds rapidly through the activation of the Rac1 within the first 3 hours. Raf is also activated during this period and blocks forgetting of a Rac1 independent form of ASM. Gene expression-based forgetting from Kdm4B/Bur activity regulates forgetting of ASM between 3–6 hours. The forgetting of consolidated ARM proceeds through the activation of Cdc42 between 3–6 hours after learning. After 6 hr memory slowly decays, but the forgetting pathways responsible are unknown (??).

Forgetting STM also depends on gene expression driven by the above DAn→NO→cGMP pathway [59] (Fig. 2). cGMP drives the co-localization of histone demethylase Kdm4B and GMP synthetase Bur at genomic sites to influence the expression of many genes including the cell adhesion protein Kek2, previously shown to regulate synaptic size [60]. Interestingly, gene expression-based forgetting was specific to 3–6 hours after learning, a timepoint following Rac1’s actions in forgetting (Fig. 3), suggestive of sequential forgetting systems working at different times after acquisition. These results corroborate NO based forgetting and link it to the expression of forgetting specific transcriptional programs.

Does forgetting of STM and LTM involve similar or different mechanisms? Interestingly, in flies, LTM, formed by repeated and spaced conditioning of odor and electric shock, is forgotten via DA→DAMB signaling but with some key differences [46]. First, unlike STM, the effect of DAn modulation on LTM is transient, as memory performance returns to normal over time. In fact, acute activation of DAn by distracting or interfering stimuli, just prior to testing, robustly and transiently suppresses LTM retrieval. Furthermore, if the DAMB receptor function is removed from MBn after LTM memory has formed, retrieval is still enhanced. The second difference is that transient forgetting involves a different DAn circuit and MBn:MBOn synapses than those involved in forgetting STM. Interestingly, LTM training creates a potentiation in odor specific MBn:MBOn synapses regulated by this DAn. This long-term memory trace was long-lasting and dependent on protein synthesis. Third, unlike the synaptic depression based STM traces mentioned above, this LTM memory trace was not significantly affected by DAn modulation. These results indicate that DA→DAMB signaling is regulating transient forgetting through modifying yet unknown synaptic connections without eroding or altering the core memory trace that is stable and persistent, which explains the re-emergence of LTM with time. However, the specific cellular and synaptic mechanism by which DAMB mediates transient forgetting and how this interferes with retrieval of this LTM memory trace remain to be elucidated. In the future, it will be interesting to see if DA based transient forgetting of LTM involves similar downstream mechanisms as forgetting of STM, including Gαq coupling, Scribble scaffolding, Rac1 activation, or ER Ca^2+^ release.

Additionally, the ability to update already consolidated LTM was recently linked to the CoRest/Rpd3 transcriptional repressor complex and gene expression in MBn [61]. Spaced conditioning *turns on* CoRest-Rpd3 mediated gene expression required for initial consolidation, but this gene expression eventually *turns off* through a compositional shift in the CoRest/Rpd3 repressor. Remarkably, if expression is not turned off, the memory remains flexible and is easily updated. However, it remains unclear what genes must be turned off to stabilize consolidated LTM, and how these gene networks intersect with DAn signaling at engram synapses.

DA plays a profound role in learning in *Drosophila* and mammals so unsurprisingly its role in forgetting is also conserved. For example, DA has been implicated to contribute to incidental forgetting. In human studies, subjects with a loss-of-function allele for catechol-*O*-methyltransferase (COMT), an enzyme that degrades dopamine, have higher levels of prefrontal cortex dopamine and higher levels of incidental forgetting [62]. At the DA receptor level, pharmacological inhibition of rat D1 dopamine receptors in the prefrontal cortex eliminates incidental forgetting while activation promotes it [63]. Additionally, injection of an antagonist to the D1 receptor in the rat hippocampus 12 hr after training enhances the persistence of 7-day food and cocaine-place conditioning memory [64]. Therefore, DA and D1 dopamine receptors are strongly implicated in mammalian active forgetting. Interestingly, mammalian DAn have also been shown to release co-transmitters like glutamate and GABA alongside DA [65, 66]. While it remains unclear if NO is also co-released in mammalian DAn, NO signaling in the mammalian cerebellum can drive either LTD [67] or LTP [68] based synaptic plasticity and thus could in theory be used for active forgetting like in *Drosophila*.

### Actin cytoskeleton remodeling and forgetting

Active forgetting relies, in part, on remodeling the actin cytoskeleton within engram synapses. The actin cytoskeleton gives neurons their shape and shrinking or growing synaptic connections requires cytoskeleton remodeling. Long-term imaging of adult cortical networks indicates that, depending on the neuron type, 15-60% of synaptic connections are dynamic; formed or eliminated over a month [69]. Importantly, experience can enhance this dynamic of synaptic connections [70] with learning driving synapse formation [71]. In addition to forming or eliminating synapses, actin cytoskeleton dynamics also orchestrate changes in existing synapse size, which has been associated with proportional changes in synaptic strength including LTP and LTD [72–74]. This modulation of synaptic connections positions cytoskeleton remodeling as a central mediator of both learning and forgetting.

**Fig. 4:**
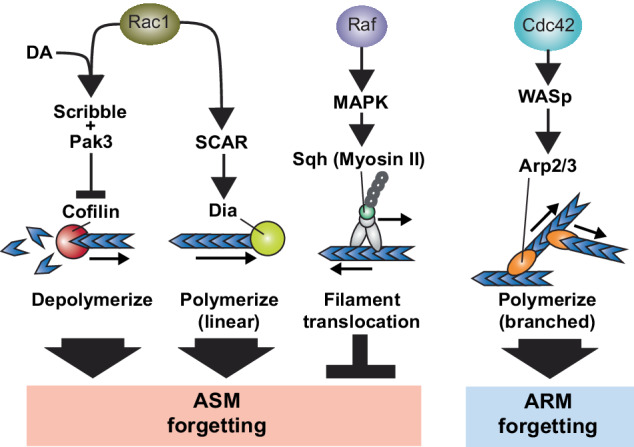
Multiple actin remodeling pathways differentially regulate forgetting of specific memory types in *Drosophila*. Actin cytoskeletal remodeling regulates the forgetting of both labile (ASM) and consolidated (ARM) memories in *Drosophila* through independent actin regulators and pathways. Small GTPase Rac1 regulates forgetting of ASM through two distinct pathways. Downstream of DA signaling, Rac1 associates with Scribble and Pak3 to inhibit Cofilin and stop actin depolymerization. In parallel, Rac1 drives linear actin polymerization via SCAR mediated Dia activity. A portion of ASM is protected from disruption by Raf phosphorylation of MAPK and subsequent activation of the Myosin II motor. This could lead to translocation of actin filaments and tension that potentially supports enlarged synaptic size required to store memory. Another small GTPase Cdc42 regulates ARM by WASp mediated activation of the Arp2/3 complex to increase polymerization of branched actin.

Actin dynamics are principally regulated by the Rho-family GTPases which include Rac1, Cdc42, and RhoA [75–77]. Recent studies in *Drosophila* indicate that both Rac1 and Cdc42 are powerful regulators of active forgetting but with interesting differences in the components of memory they regulate (Fig. 3) and their downstream pathways and cytoskeletal processes (Fig. 4). In *Drosophila*, a single trial of aversive olfactory conditioning yields at least two memory components: 1) a short-lived memory that is labile and sensitive to anesthesia (anesthesia sensitive memory, ASM), and 2) a delayed consolidated form of memory that is more long-lasting and resistant to anesthesia (anesthesia resistant memory, ARM) [9, 78, 79]. Remarkably, while Rac1 and Cdc42 are dispensable for the formation of either ASM and ARM, they independently regulate the forgetting of ASM and ARM, respectively [29, 80, 81]. Importantly, learning activates both GTPases as their phosphorylated and activated states significantly increase upon training. After learning, Rac1 is activated from 1–3 hours and regulates forgetting of ASM, whereas Cdc42 is activated by 3 hours and regulates forgetting of ARM between 3–6 hour (Fig. 3). Since both Rac1 and Cdc42 based forgetting pathways function within the MBn [29, 80, 81], they probably regulate two distinct cellular memory traces that rely on distinct actin cytoskeletal dynamics.

Rac1 and Cdc42 direct actin dynamics through downstream signaling cascades leading to modulation of actin binding proteins. Actin remodeling processes are diverse and include depolymerization and polymerization, both linear and branched (Fig. 4). As mentioned earlier, DA→DAMB based forgetting requires the scaffolding protein Scribble to coordinate DA based signaling with cytoskeletal remodeling-based forgetting. Specifically, Scribble was found to interact with Rac1 physically and genetically as well as with canonical pathway members Pak3 and Cofilin [52]. Pak family members are serine/threonine kinases that phosphorylate and inactivate the actin-binding protein Cofilin thus halting its depolymerization function [82, 83]. Expression of constitutively active forms of Cofilin within MBn blocks active forgetting, including that mediated by Scribble [29, 52]. In addition to halting depolymerization, Rac1 can also drive linear polymerization [84, 85], and genetic epistasis experiments [81] revealed that Rac1 forgetting of ASM was in part through engaging the WASP (Wiskott-Aldrich syndrome protein) related protein SCAR and downstream actin binding Formin family protein Dia, a direct mediator of *linear* polymerization (Fig. 4) [86, 87]. It remains unclear if Cofilin and Dia signaling downstream of Rac1 control independent portions of ASM or work together.

Forgetting of consolidated ARM orchestrated by Cdc42 works through a different WASP family member WASp and downstream Arp2/3 complex [81], a pathway known to nucleate branched actin filaments [88]. Future research is needed to understand why linear and branched polymerization have differential effects on labile and consolidated memory, respectively. These studies suggest that different types and temporal phases of memory have dedicated actin cytoskeletal pathways for their erasure.

Labile memories are short-lived in part because they can be disrupted by sensory experiences that drive active forgetting. However, some actin cytoskeletal regulators protect labile memories from active forgetting. Heat-stress, electric shock stress, or odor exposure cause forgetting of labile memories and this type of forgetting appears *independent* of Rac1 [89]. The investigators found that the serine/threonine kinase Raf blocks this Rac1 independent active forgetting pathway (Figs. 3 and 4). Specifically, expression of a Raf-GOF (gain of function) transgene in adult MBn enhances the stability of labile memory and protects it from disruption by post-learning experiences. Combined expression of Raf-GOF and Rac1-DN (dominant negative) stabilized memory beyond that conferred by each individual transgene and strikingly no memory decay occurred in the first 3 hour after training. Furthermore, learning itself drives Raf dependent activation of MAPK in the MBn that persists for less than 1 hour. A screen for cytoskeletal effectors identified spaghetti squash (sqh), which encodes the regulatory light chain of Non-muscle Myosin II. Myosins are multiprotein machines that bind with actin filaments and use ATP energy to create movement and force that can be used to transport cargo or, interestingly, to propel actin filaments and produce tension [90]. Zhang et al. [89] report that learning leads to increased MBn synaptic size that is normally short lived, but with increased Raf signaling (via Raf-GOF expression) this increased size is maintained longer. Overall, the authors suggest that, in *Drosophila*, learning induced Raf activation protects labile memory by maintaining structural plasticity (increased MBn synapse size) through Myosin II function. While it remains unclear which exact Myosin II function is responsible for this protection, its ability to translocate large actin filaments is consistent with maintaining enlarged synaptic size. In mammals, Myosin II’s ability to translocate actin filaments is required for its effects on dendritic spine morphology [91].

How conserved is the role for actin cytoskeletal remodeling in forgetting across the animal kingdom? At a cellular level, both GTPases Rac1 and Cdc42 are known in mammals to modulate dendritic spine morphology by controlling actin dynamics [92] and thus in theory could both regulate forgetting in mammals. While no forgetting role for Cdc42 has been demonstrated outside of *Drosophila*, Rac1 has been shown to regulate forgetting of several types of memory in mice. Rac1 activity within the hippocampus increases after contextual fear conditioning and, importantly, pharmacological inhibition of Rac1 only after training produced enhanced memory performance [93]. Furthermore, genetic manipulation of Rac1 activity up or down within the hippocampus impaired or enhanced long-term contextual fear memory, respectively, with neither altering acquisition [94]. Similar findings have been found regarding Rac1 activity in the hippocampus and forgetting of episodic memory tested by novel object recognition [95], in a social discrimination paradigm [96], and social stress induced transient forgetting [97]. However, these studies did not investigate what actin regulatory proteins are involved downstream of Rac1, thus the role of mammalian Cofilin and Dia in active forgetting is unclear.

Regarding branched polymerization and forgetting, the Arp2/3 complex was found to be involved in forgetting in the worm *C. elegans* but with an opposite role. Whereas Arp2/3 supports active forgetting in *Drosophila*, in C. elegans it appears to prevent forgetting of associative memories [98]. Finally, Myosin II has a similar cytoskeletal and synaptic role in mammals. Acute knockdown of Myosin IIb in hippocampal neurons strongly de-stabilized early LTP at these synapses without altering the initial formation of the plasticity [99]. Importantly, Myosin IIb activity was required for rapid actin filament synthesis at the synapse that is required for LTP stabilization over time. Given that LTP is associated with increased synapse size, Myosin II has a conserved function in regulating actin dynamics to maintain increased synapse size that underlies synaptic plasticity and memory.

### Glutamatergic receptor endocytosis

**Fig. 5:**
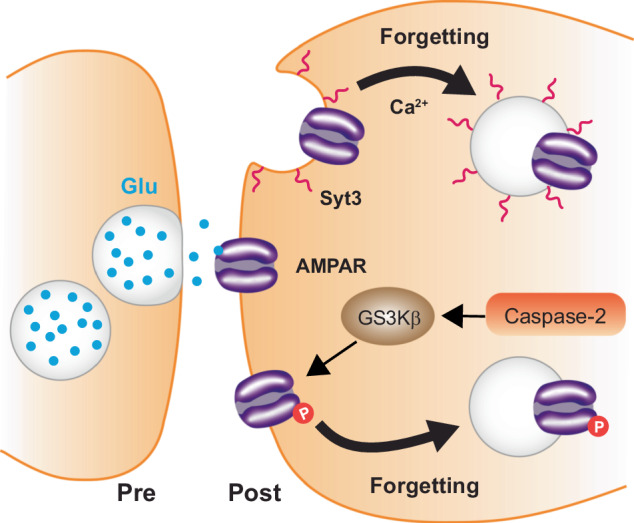
Active forgetting pathways in mammals involve AMPAR trafficking. Multiple forgetting pathways in mammals utilize glutamatergic receptor AMPAR endocytosis to modulate the strength of engram synapses. One such pathway involves the activation of Caspase-2, which subsequently increases GSK3β kinase activity. In turn, GSK3β phosphorylates AMPARs, initiating their internalization. A second and independent forgetting pathway involves Syt3 integral membrane protein and Ca^2+^ dependent internalization of AMPARs.

Like in *Drosophila*, memory engrams in mammals are encoded as modulations in strength of engram synapses, particularly of glutamatergic synapses (Fig. 5). This synaptic strength is governed by the synaptic abundance of an ionotropic glutamate receptor, AMPA (α-amino-3-hydroxy-5-methyl-4-isoxazolepropionic acid) receptor (AMPAR) [100]. The trafficking of AMPARs to the post-synaptic site following a learning event enhances synaptic strength (via LTP) and facilitates the formation of specific types of memory [101]. For example, after auditory fear conditioning, the levels of Glu2a subunit containing AMPAR in the post synapse of amygdala neurons correlate with the strength of memory [102]. Conversely, the internalization of AMPARs weakens synaptic strength, and research indicates that this drives active forgetting [103]. Several groups have shown that inhibiting the internalization of Glu2a effectively blocks the decay of LTP and the natural forgetting of memory [102, 104]. Furthermore, extinction training protocols that erase long-term fear memories do so by synaptic removal of a calcium permeable AMPAR in the lateral amygdala [105]. These studies indicate that AMPAR trafficking to and from the post synaptic membrane is a tightly regulated process to acquire and forget memories.

Multiple pathways have been shown to mediate this AMPAR trafficking. Caspase-2, a protease known for its role in apoptosis, is surprisingly also a memory suppressor gene that drives forgetting of episodic memories [106]. Reducing Caspase-2 expression slows the forgetting of spatial memory and impairs the internalization of hippocampal AMPARs. During forgetting, Caspase-2 activity increases that of GSK3β, a direct mediator of AMPAR internalization via phosphorylation. Another pathway for AMPAR internalization involves Synaptotagmin-3 (Syt3), an integral membrane protein that regulates Ca^2+^ dependent membrane recycling events. Mice lacking Syt3 fail to forget previously learned associations when presented a new learning event in the water maze due to a lack of AMPAR internalization [107]. In contrast to Caspase-2 and Syt3 pathways that drive internalization, researchers have identified that the kinase PKMζ plays a crucial role in the maintenance of AMPAR synaptic localization, thereby contributing to sustained synaptic potentiation. When PKMζ is disrupted in the dorsal hippocampus using the inhibitory peptide ZIP, mice were unable to remember the location of objects post training but could still identify the object [108]. However, there is an ongoing debate regarding the effectiveness of the ZIP peptide and PKMζ activity [109–112]. Even so, GluA2 receptor endocytosis presents an intriguing mechanism for active forgetting. Future research to unravel the signaling systems mobilized to remove the receptors will be enlightening.

### Network level regulation of forgetting

**Fig. 6:**
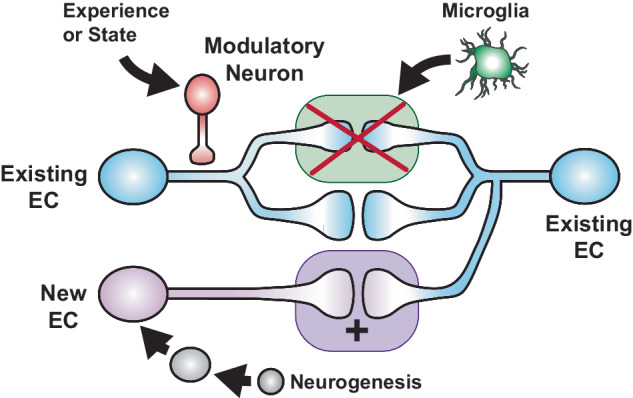
Network level regulation of forgetting. Forgetting involves multiple network level mechanisms that regulate engram cell (EC) synapses. An existing memory engram is stored across engram cells and their synaptic connections (blue). These EC are modulated by external circuits (red) that respond to sensory experience or internal states and regulate EC synaptic strength and thus regulate forgetting. Additionally, neurogenesis drives the creation of new engram cells (purple) that compete for synaptic inputs and outputs with existing engram cells storing pre-existing memories. Here, “+” indicates the recent addition of a synapse between new EC and existing EC. Finally, weaker engram synapses are targeted for phagocytosis and elimination by microglia (green).

The active forgetting mechanisms discussed above involve cellular signaling within engram cells to modulate their synaptic strength and accessibility of memories. However, these mechanisms are further tuned by factors external and internal to the animal, including environmental experience, physical activity, arousal, sleep, and neurogenesis. These factors modulate forgetting at the network level by regulating neural circuits that synapse onto engram cells, synaptic competition between engram cells, and microglial remodeling of synapses (Fig. 6). Thus, non-engram brain cells provide the platform for brain-wide and network level information to access engram synapses and regulate cellular forgetting pathways.

Sensory experience can modulate the rate of forgetting across the animal kingdom. As discussed above in *Drosophila*, sensory experiences like heat and electric shock stress, odor exposure, or even new learning can disrupt labile memory. Given that DAn are responsive to most of the sensory experiences above [38, 43] they are positioned as ideal conduits for sensory experience to modulate forgetting. Stimulating any of several different DAn circuits after learning is sufficient to simultaneously drive learning and forgetting of specific memory engrams [37, 49, 50]. Experiences seemingly unrelated to the original training can also drive forgetting via DAn. For example, mechanical stimulation induced arousal and locomotion accelerates forgetting of memories via enhancing the activity of the same DAn that encoded them [113]. Thus, DAn driven active forgetting mechanisms provide a biological explanation for interference-based forgetting. Finally, in worms, the presence of food activates forgetting of odor adaptation memory in engram neurons through the secretion of forgetting signals from separate sensory neurons [114]_._

Housing in environments enriched with increased levels of sensory, cognitive, and motor stimulation can modulate synaptic plasticity in rodents [115] and enhance cognitive flexibility in mice [116]. On the other hand, social isolation in an impoverished environment enhances forgetting of social recognition memories in mice via increasing Rac1 activity in the hippocampus [96]. So, the direction by which the environment modulates forgetting varies with the scenario and likely the relatedness between the training and post-training stimuli and context.

Internal brain states, like sleep, also modulate active forgetting mechanisms. Sleep consists of several stages with diverse effects on neural activity and has a well-established role in enhancing memory after learning across the animal kingdom, including in humans and fruit flies [113, 117–124]. In flies, sleep is associated with diverse changes in the activity of specific neural circuits across the brain, including DAn [113, 117]. The underlying mechanisms of sleep-dependent memory enhancement are still being defined and are diverse. One mechanism is that sleep enhances memory consolidation [118–121]. This is supported by studies showing that engram cells in the hippocampus are reactivated during sleep to promote memory consolidation [122, 123]. Another possible mechanism, not mutually exclusive with the first, is that sleep and rest inhibit active forgetting of labile memory, potentially protecting nascent memory traces from interference so they can be consolidated [113, 124]. Interestingly, the *Drosophila* DAn that drive forgetting of labile memory have a baseline level of ongoing activity that steadily releases DA onto the engram cell synapses and this increases with arousal and locomotion [48, 51, 113, 125, 126]. Efficient release of DA during this ongoing activity and forgetting requires the memory suppressor and cytoskeletal protein Sickie in presynaptic terminals of DAn [30, 126]. Importantly, Berry et al. [113] showed that sleep efficiently blocks this ongoing dopamine based forgetting signal and increases memory retention. This provides a biological explanation for how sleep retroactively facilitates memory by protecting it from interference. In contrast to enhancing memory retention, sleep has also been proposed to *drive* active forgetting in at least two ways. First, sleep leads to synaptic downscaling that broadly reduces synapse strength and thus theoretically could degrade engrams [127–129]. Second, a recent study identified a hypothalamic neural circuit that is active during sleep and inhibits hippocampal neuron activity to drive active forgetting [130]. Altogether, sleep modulates the persistence of memory in multiple ways to balance the consolidation of important memories and active forgetting of unimportant ones.

Additional non-engram neural circuits have been identified that drive active forgetting but have yet to be connected to internal or external factors. In *Drosophila*, two MB extrinsic circuits, a DAn and a MBOn, independently regulate active forgetting but are dispensable for learning [51]. Most interestingly, these forgetting circuits have a restricted forgetting function, regulating time-based forgetting without influencing interference-induced forgetting. Both circuits provide pre-synaptic input to many target neurons within specific MBn:MBOn synaptic regions [51, 131] and could in principle regulate engram synapses directly or indirectly. It will be interesting to find out what network level factors, internal or external, regulate these circuits and modulate forgetting.

In addition to circuit and cellular mechanisms that modulate *existing* engram synapses, the creation and addition of entirely new engram cells and their synaptic connections also drives active forgetting in mammals (Fig. 6 bottom). Throughout life, the dentate gyrus neurons in the hippocampus are continuously added through the process of neurogenesis. These new engram cells compete for synaptic inputs and outputs with older engram cells, including presumably those with stored information. Thus, neurogenesis has been proposed as an intriguing mechanism to forget old memories stored in old engram cells [132, 133]. Indeed, increasing hippocampal neurogenesis after learning accelerates forgetting, while reducing it attenuates it in mice [134–136]. At a synaptic level, suppressing neurogenesis creates a longer-lasting hippocampal LTP [137] and increased conditions that drive neurogenesis lead to a faster LTP decay [138]. The rate of neurogenesis depends on many factors mentioned above including physical activity and environmental enrichment [134–136]. Interestingly, neurogenesis might also be coordinated with the engram cellular mechanisms discussed above including Rac1. Rac1 is not required for basal neurogenesis but is required for learning evoked increases in neurogenesis [139]. If learning induces both Rac1 expression in engram synapses and neurogenesis, it opens the possibility that these forgetting systems are coordinated. However, it remains unclear if and how neurogenesis alters synaptic physiology of specific engram cells for a specific memory. Further experiments using tagged engram cells and their synapses will be required to measure the direct effects of neurogenesis on an engram.

Finally, neurons are only one type of brain cell that can regulate memory. Mammalian neuronal synapses are surrounded by the projections of small glial cells called microglia. Microglia recognize synapses that express specific complement proteins, engulf, and eliminate them in an activity dependent manner [140, 141]. A recent study demonstrated that natural forgetting involves microglia-dependent mechanisms [142]. Mice trained in contextual fear conditioning had reduced forgetting when microglia where pharmacological or genetic depleted after learning. Additionally, reducing activity of tagged engram cells in mice after learning led to forgetting that was entirely dependent on microglia. Given that microglia target weak synapses over strong [141], one possibility is that less active engram cell synapses are pruned by microglia leading to selective forgetting of infrequently used memories (Fig. 6 top right).

## Summary and perspectives

A major conclusion offered here is that the brain has the inherent capacity and flexibility to forget information though dedicated networks and molecular cascades. This capability is not a passive process, but is active since pathways like Rac1, Cdc42, and NO are triggered by learning itself. Thus, active forgetting, along with acquisition, consolidation, and retrieval, are equally important parts of the brain’s memory management system. Across the animal kingdom, a common end “goal” of these pathways is to modulate engram cell synaptic strength, either through DA and NO mediated synaptic modulation as in flies, or through excitatory glutamatergic receptor endocytosis, neurogenesis, and microglia in mammals (Figs. 2, 5, 6).

A second conclusion we offer is that memory is both formed and forgotten in layers. The brain employs many molecular and cellular mechanisms to form robust and salient memories. We speculate that many of these mechanisms may have their own independent forgetting mechanisms to reverse the changes made to the neuron’s structure and/or function. It follows that distinct forgetting pathways will exist for memories of different temporal phases (STM, LTM, etc) and kind (aversive, appetitive, episodic, motor, etc.). For instance, Rac1 regulates memory decay prior to that of Kdm4B/Bur and Cdc42, but forgetting continues beyond these time domains. This suggests that remote memory may be slowly forgotten by mechanisms independent of Rac1, Kdm4B/ Bur, and Cdc42 (Fig. 3). Additionally, reward memory is insensitive to Rac1-Dia based active forgetting in *Drosophila*, suggesting distinct pathways depending on valence [143].

A third conclusion is that cellular forgetting in engram cells is heavily influenced by non-engram brain cells and circuits and by newly born engram cells. This feature allows internal brain states and external experience to fine-tune active forgetting within engram cells. For example, the internal state of sleep can decrease active forgetting signals from DAn to protect nascent memory and facilitate consolidation. Furthermore, enriched environments and physical exercise increase adult neurogenesis of future engram cells that will outcompete older engram cells for synaptic inputs and outputs and drive the forgetting of old unused memories. Interestingly, there is evidence that forgetting mechanisms at different levels of complexity work together. Learning induced neurogenesis requires Rac1 [139] and microglial remodeling [142] suggesting that cellular forgetting pathways may be coordinated with neurogenesis and microglial remodeling. There are probably many other network-level active forgetting mechanisms that will be uncovered from future genetic and circuit level screening in model organisms.

Finally, we offer the perspective that alterations in active forgetting participate in the pathophysiology of human brain disorders. In addition to the direct evidence for impairments in intentional and incidental forgetting in individuals with neuropsychiatric diseases discussed above, evidence has emerged suggesting that some types of autism spectrum disorder in both human subjects and mouse genetic models are associated with reversal learning or active forgetting deficits [144, 145]. Moreover, recent research has uncovered a role of Rac1 in the accelerated forgetting associated with neurodegeneration in Alzheimer’s disease (AD) [146, 147]. Thus, dissecting the mechanisms of active forgetting promises a wealth of information and a renewed understanding on how a normal and abnormal brain manages the incomprehensible amount of information that it processes daily. It also offers the promise of identifying new molecular targets for cognitive enhancers that could fine-tune forgetting. Although the neuroscience mechanisms for active forgetting are just beginning to emerge with some clarity, it is not too early to imagine how current and future mechanisms yet to be discovered are involved in neuropsychiatric diseases and brain disorders that affect memory retention.
